# Assessing the Impact of Uterine Artery Doppler and Low-Dose Aspirin on Fetomaternal Outcome: A Prospective Study in Low-Risk Pregnant Women in Western Part of India

**DOI:** 10.7759/cureus.42515

**Published:** 2023-07-26

**Authors:** Rashmi B Patel, Ajay K Patel, Manish Y Machave, Sunita R Tandulwadkar, Puja A Lodha, Himel Mondal

**Affiliations:** 1 Obstetrics and Gynecology, All India Institute of Medical Sciences, Deoghar, Jharkhand, IND; 2 Anatomy, All India Institute of Medical Sciences, Deoghar, Jharkhand, IND; 3 Obstetrics and Gynecology, Ruby Hall Clinic, Pune, IND; 4 Fetal Medicine and Fetal Therapy, Ruby Hall Clinic, Pune, IND; 5 Physiology, All India Institute of Medical Sciences, Deoghar, Jharkhand, IND

**Keywords:** antenatal care, ultrasound, colored flow doppler ultrasound, hypertension pregnancy induced, aspirin, umbilical artery pulsatility index, fetal growth retardation, pregnant women, pre-eclampsia, pregnancy

## Abstract

Introduction

Fetal growth restriction (FGR) and pregnancy-induced hypertension (PIH) are significant and clinically relevant complications observed in many pregnancies. Early prediction of these complications may be possible through the assessment of the umbilical artery pulsatility index (UAPI). However, its utility in routine practice for otherwise normal pregnancy needs further exploration in India.

Objectives

This study aimed to evaluate the potential benefits of incorporating UAPI for the timely use of low-dose aspirin in preventing FGR and PIH in a tertiary care hospital in the western part of India.

Methodology

A prospective study was conducted involving 64 low-risk (i.e., not having any feature of high-risk pregnancy) pregnant women selected from routine antenatal care outpatient departments over a period of two years. All women underwent uterine artery Doppler examination during the 11-13+6 weeks of pregnancy and those who had high UAPI received low-dose (150 mg) aspirin till the 35th week. The incidence of FGR and PIH was analyzed and compared between high UAPI and normal UAPI pregnancy.

Results

A total of 64 pregnant women with a mean age of 27.11±4 years participated in the study. Among the women, eight (12.5%) were found to have high UAPI and were put on aspirin. Among those eight women, two developed PIH. In the normal UAPI group, nine (16.07%) developed PIH (p-value = 0.62). FGR was found in one case among the eight who received aspirin and in eight cases among the 56 who had normal UAPI (p-value > 0.99).

Conclusion

The study concluded that despite having normal UAPI, women categorized as low-risk may develop PIH and FGR. Hence, the routine use of UAPI should be investigated in further cohort studies using a large sample to draw a generalizable conclusion for the Indian population.

## Introduction

Placental circulation plays a critical role in supporting fetal development and ensuring a healthy pregnancy. The establishment of this circulation occurs on approximately day 17 post-fertilization [1]. The development of uteroplacental vessels follows a two-wave process. The first wave occurs 12 weeks post-fertilization and involves the invasion and remodeling of spiral arteries up to the decidua-myometrium boundary. The second wave occurs between 12 and 16 weeks and involves some invasion of spiral artery intra-myometrial segments. Doppler analysis can show impaired invasion during these waves [2].

One of the key parameters used in Doppler assessment is the umbilical artery pulsatility index (UAPI) [3]. This index, derived from systolic and diastolic velocities, provides a quantitative measure of resistance to blood flow. Changes in UAPI can indicate alterations in uteroplacental circulation and may predict adverse pregnancy outcomes such as pregnancy-induced hypertension (PIH) and fetal growth restriction (FGR) [4,5].

Low-dose aspirin (acetylsalicylic acid) has been widely used to improve pregnancy outcomes, specifically in preventing PIH and FGR [6]. The administration of daily oral doses ranging from 50 to 150 mg effectively inhibits platelet thromboxane A2 biosynthesis, thus mitigating potential complications. A study in Iraq found that a screening test using Doppler investigation of the uterine arteries at 16 weeks or more in low-risk pregnant women is useful and that complications can be reduced by early administration of a modest dosage of aspirin [7]. However, a study in France showed that there is no value of UAPI in routine care for pregnant women [8].

In this context, this prospective study aimed to investigate the role of UAPI and low-dose aspirin intervention in improving fetomaternal outcomes in low-risk pregnant women in a city in India.

## Materials and methods

Type and setting

This was a prospective study conducted in the Department of Obstetrics & Gynecology and Fetal Medicine at Ruby Hall Clinic, a Research and Referral hospital in Pune, Maharashtra, India. The study was conducted from June 2017 to May 2020.

Participants

A total of 64 antenatal women were selected for the study based on specific inclusion and exclusion criteria. The inclusion criteria encompass singleton pregnancies, first-time pregnant women (primi gravidae), and a gestational age ranging from 11 to 13+6 weeks. The gestational age was determined using an early first-trimester ultrasound examination, specifically measuring the crown-rump length (CRL) and relying on a reliable medical history. On the other hand, the exclusion criteria involve preexisting medical conditions such as gestational diabetes mellitus (GDM) and PIH, as well as multiple pregnancies during recruitment. Women aged over 35, those with a body mass index (BMI) equal to or exceeding 25 kg/m2, those with a family history of pre-eclampsia, or those with documented congenital anomalies were excluded from the study. By adhering to these criteria, we ensured to include apparently low-risk pregnancies.

UAPI method

To acquire a midsagittal slice of the uterus and cervical canal, a 5- or 3.5-MHz curvilinear transabdominal transducer was employed. After that, the transducer was pushed laterally until the paracervical arteries were visible. The uterine arteries were identified as aliasing vessels down the side of the cervix using Doppler [9,10]. The pulsed wave with a Doppler sampling gate was set at 2 mm and a modest angle of insonation (30°), and flow velocity waveforms from the ascending branch of the uterine artery near the internal os were obtained. The pulsatility index was calculated when three successive identical waveforms were acquired.

Data collection

Comprehensive history-taking and physical examinations were conducted for all participants. UAPI measurements were performed between 11 and 13+6 weeks of gestation, along with nuchal translucency and nasal bone scans. The UAPI was evaluated in a cohort of 64 low-risk pregnant women. Among the pregnant women with raised UAPI at 11 to 13+6 weeks, a low dose of aspirin (150 mg/day) was introduced at or after 12 weeks of gestational age till 35 weeks. Follow-up was carried out until 36 weeks of gestation for all recruited women. Final fetal and maternal outcomes were assessed in late pregnancy through routine check-ups, including blood pressure monitoring after 20 weeks. Ultrasound scans were performed between 32 and 36 weeks to assess FGR.

Statistical tests

Statistical analysis was performed using GraphPad Prism 9.5.0 (GraphPad Software Inc., GraphPad Software, Boston, MA, USA). The results for categorical data were presented as numbers and percentages. Fisher’s exact (as frequency is below 5) test was used to statistically compare the distribution of categorical variables. A p-value of less than 0.05 was considered statistically significant.

Ethics

The study was conducted in accordance with ethical guidelines laid by the WMA Declaration of Helsinki, updated in 2013. The study was approved by the Institutional Review Board of Ruby Hall Clinic (number: IRB/2020/212351). All the research participants were recruited after getting a voluntary written informed consent form.

## Results

A total of 64 pregnant women with a mean age of 27.11±4 years participated in the study. The height, weight, BMI, heart rate, and blood pressure of the participants are shown in Table 1.

**Table 1:** Height, weight, BMI, heart rate, and blood pressure of the participants mmHg, millimeter of mercury; bpm, beats per minute

Parameter	Mean	Standard deviation	Range
Age (years)	27.11	4	22–34
Height (cm)	156.33	4.05	151–168.6
Weight (kg)	53.92	3.65	48–62
BMI (kg/m^2^)	22.08	1.51	18.7–24.9
Heart rate (bpm)	85.81	8.96	67–104
Systolic blood pressure (mmHg)	114.95	3.77	106–119
Diastolic blood pressure (mmHg)	73.2	3.78	67–78

Of the 64 women screened, eight (12.5%) had high UAPI, which is a risk factor for developing pre-eclampsia and FGR; the remaining 56 (87.5%) had normal UAPI.

The eight women who had high UAPI received low-dose aspirin (150 mg/day) for up to 35 weeks, and two of them developed PIH. However, among the 56 women who had normal UAPI, nine (16.07%) women developed PIH. The distribution in the contingency table is shown in Table 2.

**Table 2:** Pregnancy-induced hypertension in two groups according to UAPI UAPI, umbilical artery pulsatility index Fisher's exact test p-value = 0.62

Pregnancy-induced hypertension	UAPI		Total
	High	Normal	
No	6	47	53
Yes	2	9	11
Total	8	56	64

The FGR was observed in one woman who has been on aspirin therapy. Of those who had normal UAPI, eight had FGR, as shown in Table 3.

**Table 3:** Fetal growth retardation in two groups according to UAPI UAPI, umbilical artery pulsatility index Fisher's exact test p-value > 0.99

Fetal growth retardation	UAPI		Total
	High	Normal	
No	7	48	55
Yes	1	8	9
Total	8	56	64

## Discussion

In recent years, the measurement of UAPI in the first trimester has gained increasing usage. Various techniques and impedance indices have been employed to evaluate the association between uterine artery Doppler measurements and adverse pregnancy outcomes [11]. In the current study, the first-trimester UAPI measurement could not show any significant benefit for identifying PIH or FGR.

Prajapati and Maitra in a study in India concluded that UAPI in the second trimester is a good screening technology for identifying high-risk pregnancies in women who may be closely monitored for better maternal and neonatal outcomes. This test is more effective when paired with a history of pre-eclampsia [12]. Barati et al. also support that the uterine artery Doppler examination at 16-22 weeks of gestation may be a suitable method for detecting pregnancies at greater risk of pre-eclampsia and undersized fetuses for gestational age [13]. In a study in India, Handa and Pujar found that UAPI has very low sensitivity for predicting eclampsia and this limits the utility of this test alone [14]. Supporting this, Pedroso et al. suggested that the Doppler alone has very low applicability in predicting PIH or FGR [15]. The present study showed that even after predicting the risk and starting the low-dose aspirin, there was no significant change in outcome. This finding highlighted that might be a result of advanced gestational age at initiation of low-dose aspirin.

The lack of significant benefit observed in our study could be attributed to several potential causes. The timing of measurement might not accurately capture the development of these conditions during the early stages of pregnancy. Physiological variability in uterine blood flow and vascular adaptations during this period could impact the UAPI values, making it challenging to establish a consistent cutoff for identification [16]. Additionally, the multifactorial nature of PIH and FGR, influenced by various maternal and environmental factors, may require complementary diagnostic tools beyond UAPI measurement [17,18]. The sample size, study design, measurement technique, and interoperator variability might also contribute to the observed findings. Further research is needed to validate these results and explore alternative approaches for the early identification of PIH and FGR during the first trimester.

In the study, we found that despite having normal UAPI, 16.07% developed PIH. This finding is corroborative to the Indian population as the incidence of PIH in hospital practice in India varies from 5% to 15% [19]. That study found FGR in 12.29% of pregnancies, which is between the incidence in higher socioeconomic groups (3-9%) and low socioeconomic groups (30%) [20,21].

Limitations of the study

Several limitations should be considered when interpreting the findings of this study. The small sample size, consisting of 64 low-risk pregnant women (eight of them received low-dose aspirin) from a single tertiary care hospital in a specific geographic region, may limit the generalizability of the results. The absence of a control group and randomization in the study design prevents establishing a causal relationship between UAPI measurement and the occurrence of FGR and PIH. The study's focus on UAPI and low-dose aspirin as the sole interventions disregards other potential factors influencing these outcomes. Additionally, the study did not assess long-term outcomes or evaluate the sustained effects of low-dose aspirin beyond the 35th week of pregnancy. The study's limitations emphasize the need for larger-scale studies with diverse populations and more comprehensive methodologies to better understand the role of UAPI measurement and low-dose aspirin in preventing FGR and PIH.

## Conclusions

Contrary to prevailing assumptions, the present investigation demonstrates that women with normal UAPI measurements can still develop PIH and FGR. This unexpected outcome challenges the reliability and efficacy of UAPI as a standalone indicator for these complications. These findings prompt a paradigm shift in the field of obstetrics and call for renewed efforts to improve the precision and efficacy of diagnostic tools for the early identification of PIH and FGR.
